# A case report of eosinophilic myocarditis and a review of the relevant literature

**DOI:** 10.1186/s12872-015-0003-7

**Published:** 2015-02-26

**Authors:** Haiying Li, Zhenyu Dai, Binqiao Wang, Weijian Huang

**Affiliations:** Department of Cardiology, The First Affiliated Hospital of Wenzhou Medical University, Wenzhou, 325000 China

**Keywords:** Eosinophils, Antituberculosis drugs, Myocarditis, Corticosteroids

## Abstract

**Background:**

Eosinophilic myocarditis (EM) is a relatively rare condition that may result from parasitic infections and allergic disease. Antituberculosis drugs may lead to focal myocardial infiltration by eosinophils (eosinophilic myocarditis). Symptoms may be severe, and, lead to rapidly-fatal outcomes. Early diagnosis and high-dose corticosteroids are the cornerstone of treatment, and, may lead to restoration of cardiac function with full recovery.

**Case presentation:**

We report a case of eosinophilic myocarditis secondary to eosinophilia caused by antituberculosis drugs with markedly elevated ECP, focal eosinophilic infiltration in CMR imaging and endomyocardial biopsy. Finally, high-dose corticosteroids were used to reverse the cardiac injury and to improve the clinical outcome.

**Conclusion:**

Antituberculosis drugs can cause eosinophilic infiltration of, and damage to, the myocardium leading to rapid progression of the clinical symptoms. Myocardial biopsy is helpful in diagnosing the disease in the early stages and high-dose corticosteroids effectively improves the prognosis of this disease.

## Background

Eosinophilic cells (EC) constitute 0% to 7% (0.05–0.45 × 10^9^/L) of normal leukocytes; however, the exact function of this cell type remains to be fully elucidated. ECs participate in normal immune responses although abnormal increases in this population can cause tissue damage. ECs contain cytotoxic granules with an array of cationic proteins including eosinophilic cationic protein (ECP), eosinophil peroxidase, and eosinophil-derived neurotoxin as well as numerous RNases, and produce reactive oxygen species such as peroxidase, elastase, cytokines. Following activation of ECs by an immune stimulus, these substances are released by degranulation to mediate tissue damage and dysfunction. ECs have surface proteins that specifically bind IgE complexed with antigen to induce phagocytosis and degranulation [1].

A definitive diagnosis of eosinophilic myocarditis(EM) [2] eosinophilic myocarditis is made on the basis of two criteria: fulfillment of the four criteria for diagnosis of carditis (ECG or Holter or stress test features, elevated troponin T (TnT) or troponin I (TnI) markers of myocardiocytolysis, functional and structural abnormalities in cardiac imaging and cardiac magnetic resonance (CMR) tissue characterization and a histological diagnosis based on the type of inflammatory cell infiltrate. EM is a relatively rare condition that may occur in a variety of settings including parasitic infections and allergic disease. The best known form of this disease is the Loeffler endomyocardial fibrosis, which occurs as a major complication of idiopathic hypereosinophilic syndrome. Extensive myocarditis can be caused by parasites, leading to high mortality, and in some cases is caused by drug reactions and allograft rejection in heart transplantation. In addition, Churg-Strauss Syndrome can produce myocardial granulomas and extensive myocardial lesions can cause heart failure, progressing to dilated cardiomyopathy (DCM) in some patients. Among the inflammatory cells that infiltrate the cardiac tissue, which is secondary to these types of diseases, the damage caused by EC is significant [3]. The aim of this study was to increase recognition of EM and the use of endomyocardial biopsy (EMB)The use of endomyocardial biopsy(EMB), which is the gold standard in Europe, for effective identification of cases caused by EC infiltration in response to allergic reactions to antituberculosis drugs. To address this issue, we present a clinical case of myocardial damage apparently secondary to eosinophilia and conducted a comprehensive investigation of the relevant case presentations, diagnosis, and treatment reported in the literature to improve early diagnosis for the timely initiation of clinical treatment of myocardial lesions caused by eosinophilia.

## Case presentation

On March 6, 2012, a 43-year-old Chinese businessman with a 5-year medical history of hypertension presented with recurrent chest pain and chest tightness commencing six months prior to his admission to the Cardiology Department of the First Affiliated Hospital of Wenzhou Medical University. The patient denied any history food and drug allergies and endemic water exposure.

On January 16, 2012, the patient had been admitted to our hospital for treatment of fever, persistent severe headaches with chills, nausea and vomiting. On admission, his temperature was 39°C, he had nuchal rigidity and was Kernig sign positive but Brudzinski sign negative, with other components of his physical examination found to be non-contributory. Laboratory tests of CSF showed the following: WBC, 146/μL; LY, 91%; Pandy test positive; Pro, 2,767 mg/L; Glu, 1.9 mmol/L; Cl^−^ 106, mmol/L. Intracranial infection was clinically suspected, with tuberculous meningitis primarily considered; therefore, the patient was administered the standard tuberculosis control regimen of isoniazid, rifampicin, pyrazinamide and ethambutol (HRZE). During the course of treatment, however, rifampicin (RFP) was discontinued due to impaired hepatic function. The patient was then started on diammonium glycyrrhizinate treatment, which gradually improved the hepatic function. Eventually, the patient’s condition stabilized, his temperature normalized and the symptoms were markedly resolved. He was discharged 50 days later with continued HZE medication.

On March 6, 2012, the patient was again hospitalized complaining of persistent chest tightness and chest pain which progressed dramatically. His temperature was 38.6°C with no chills, coughing or dyspnea at that time. Chest CT imaging indicated a small amount of pericardial effusion but no pulmonary infiltration. Laboratory data revealed the following: platelet count, 36 × 10^9^/L; eosinophils 3.0 × 10^9^/L; Tn, 7.23 ng/mL; creatine kinase-MB (CK-MB), 44 U/L.

**Figure 1:**
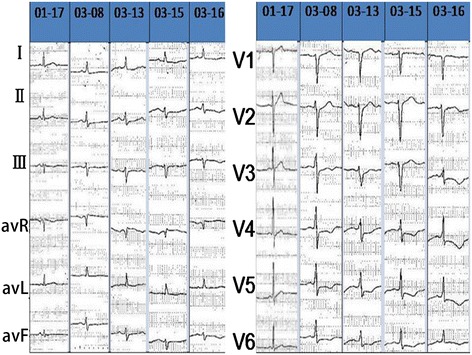
**The dynamic changes of ECG.** T waves in leads V3–V6 observed in ECG were flat or inverted.

After hospitalization, the patient’s blood pressure decreased gradually to approximately 90/60 mmHg (1 mmHg is equal to 0.133 kPa) with dopamine treatment (3–8 μg/kg/min). His urine volume remained normal and his extremities were warm. During the first three days in hospital, the patient’s myocardial enzymes continued to elevate, with Tn rising to 50.0 ng/ml, CK reaching 410 U/L and CK-MB increasing to 52 U/L. Routine blood tests showed marked EC elevation (5.0 × 10^9^/L). In addition, flat or inverted T waves in leads V3–V6 were observed in ECG (Figure 1).

During the diagnostic investigation of etiology, additional laboratory analyses including repeated blood cultures, blood tumor series and autoimmune series, provided no evidence of hematogenous infection or autoimmune diseases. Bone marrow aspiration cytology showed myeloproliferation, elevated ECs and megakaryoblast hyperplasia with disordered maturation. There were no abnormal cells and hematopathy was excluded. Comparison with the patient’s previous CSF results showed that his infection was relatively controlled.

On March 12, 2012, echocardiography showed that the patient had normal movement of the ventricular wall but impaired diastolic function (left ventricular end diastolic diameter (LVEDD), 47 mm; interventricular septum thickness (IVS), 12 mm; left ventricular ejection fraction (LVEF), 50.8%).

**Figure 2:**
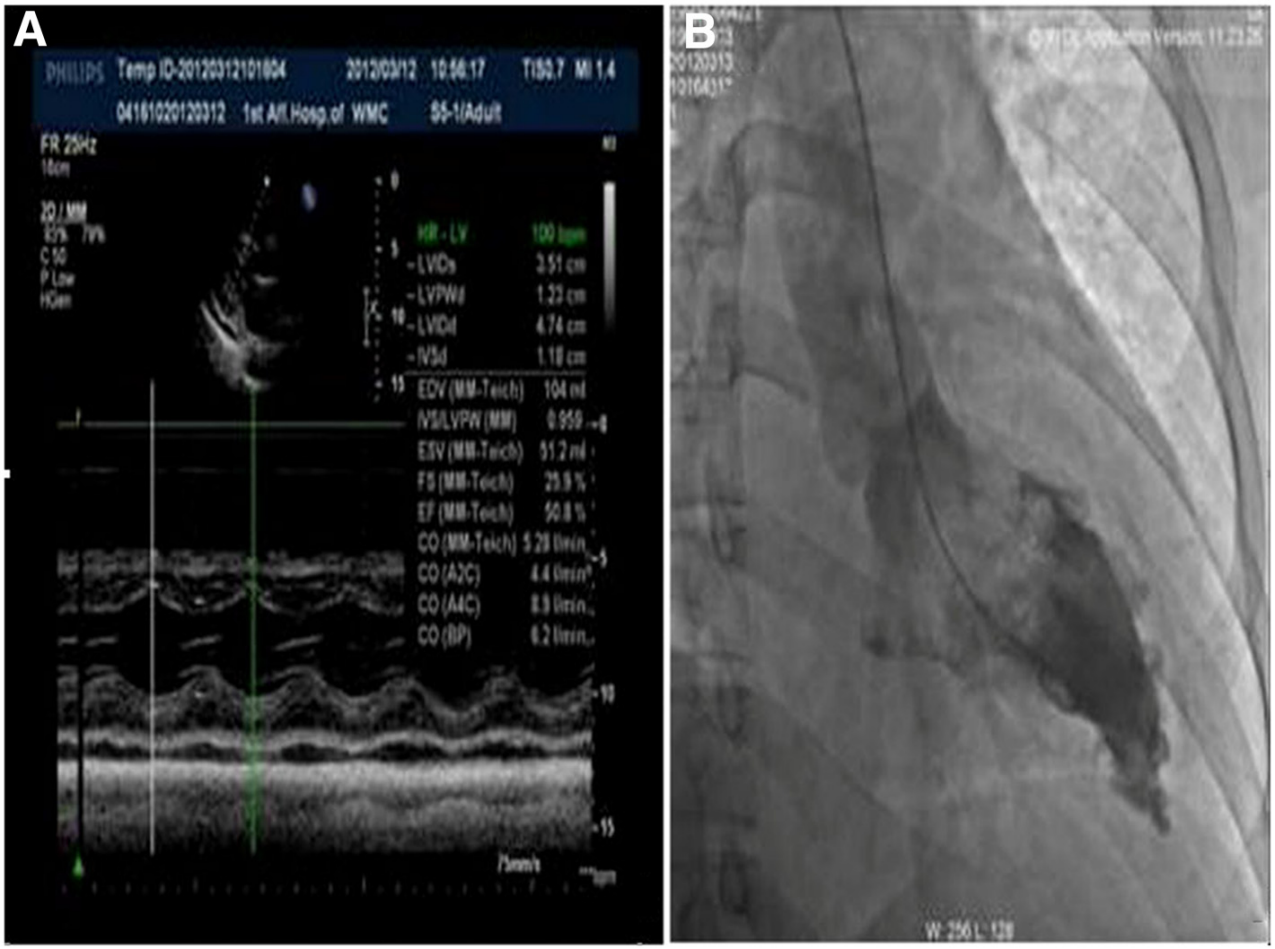
**Echocardiography and left ventricular angiography. A**. Echocardiography revealed normal movement of the ventricular wall and deteriorated diastolic function (left ventricular end diastolic diameter (LVEDD), 47 mm; interventricular septum thickness (IVS), 12 mm; left ventricular ejection fraction (LVEF), 50.8%). Coronary angiography showed that there was no stenosis or obstructive lesions in the coronary artery; **B**. Left ventricular angiography showed thickening of the apical ventricular membrane.

On March 13, 2012, coronary angiography showed that there was no stenosis or obstructive lesions in the coronary artery. Left ventricular angiography revealed thickening of the apical ventricular membrane (Figure 2). Swan-Ganz catheterization indicated normal systolic function with disordered cardiac diastolic function, which recovered substantially after blood pressure stabilization treatment and adequate fluid infusion (Table 1).

**Table 1:** **Swan-Ganz catheter measurements**

	**CO L/min**	**CI ** L/min*m ^**2**^	**CVP mmHg**	**PCWP mmHg**	**PA mmHg**	**SVR DS/cm**	**SVRI DS/cm**	**PVR ** **DS/cm** ^**2**^	**PVRI ** DS*m ^**2**^ **/cm** ^**2**^
03.13	4.7	2.8	5	18	20	1116	1752	36	57
16:00									
03.13	3.75	2.4	9	18	20	1173	1798	43	65
19:00									
03.13	3.98	2.6	7	15	18	1186	1819	141	216
21:00									
03.14	3.76	2.4	11	22	24	1277	2005	64	100
14:30									

**Figure 3:**
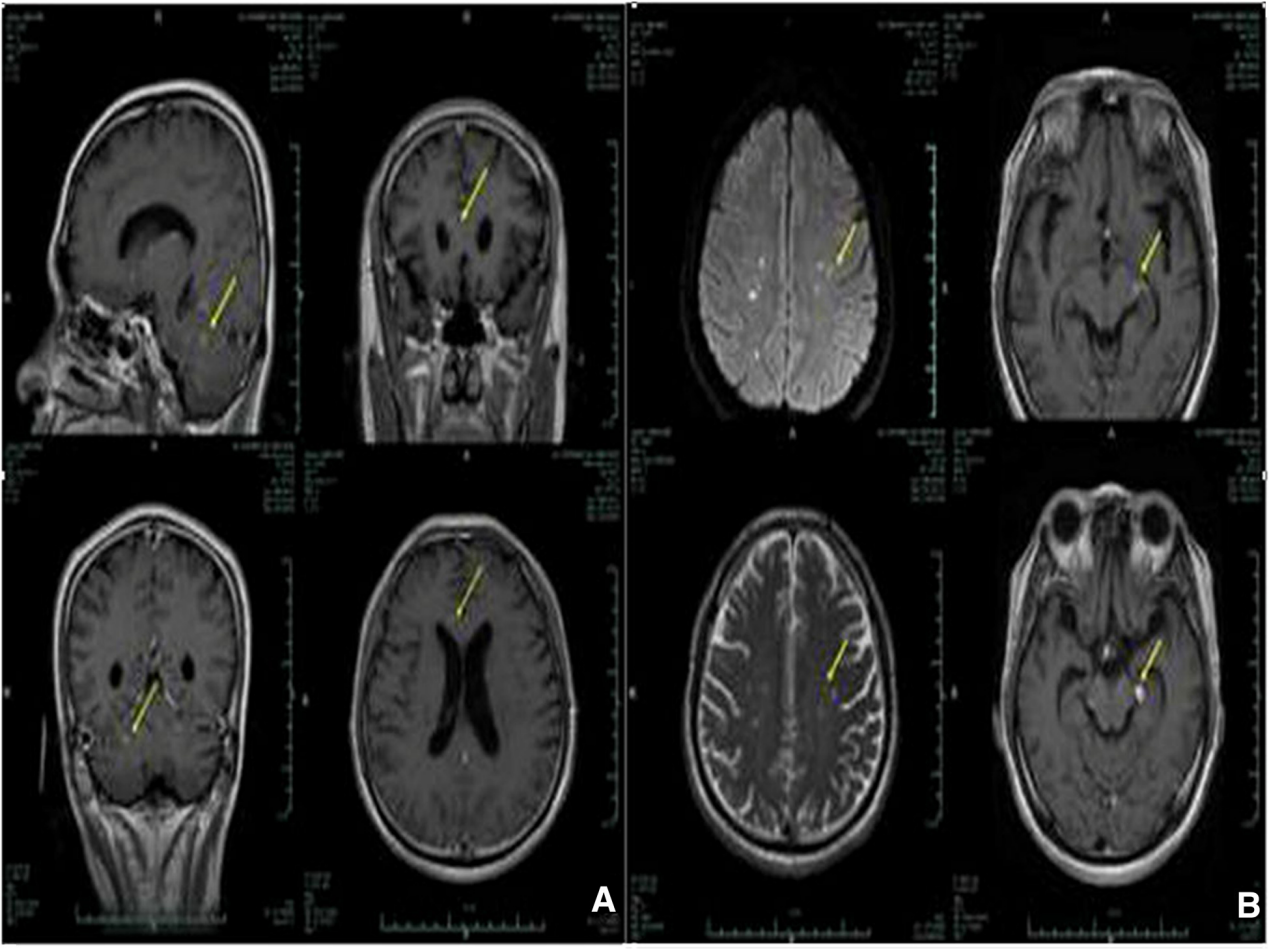
**Head MRI. A**. Head MRI taken on February 24; **B**. Head MRI taken on March 13. Comparison of the results of the head MRIs revealed the continued presence of meningeal infiltration and no obvious improvement in the parenchymal nodules in the brain.

**Figure 4:**
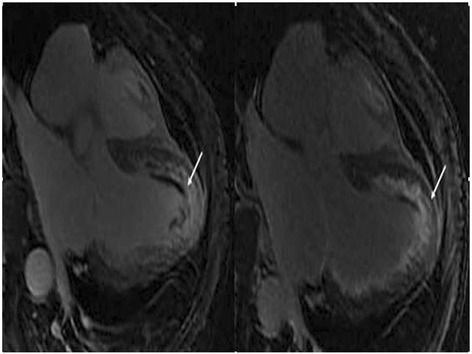
**Cardiac magnetic resonance (CMR) imaging.** CMR imaging showed significant enhancement of an irregular stripe signal after left ventricular subendocardial enhancement in the delay period and abnormal enhancement of patch and stripe signal in the ventricular septum.

Repeated head MRIs revealed the continued presence of meningeal infiltration and no obvious improvement in the parenchymal nodules in the brain (Figure 3). Cardiac MRI showed significant enhancement of an irregular stripe signal after left ventricular subendocardial enhancement in the delay period and abnormal enhancement of patch and stripe signal in the ventricular septum (Figure 4).

**Figure 5:**
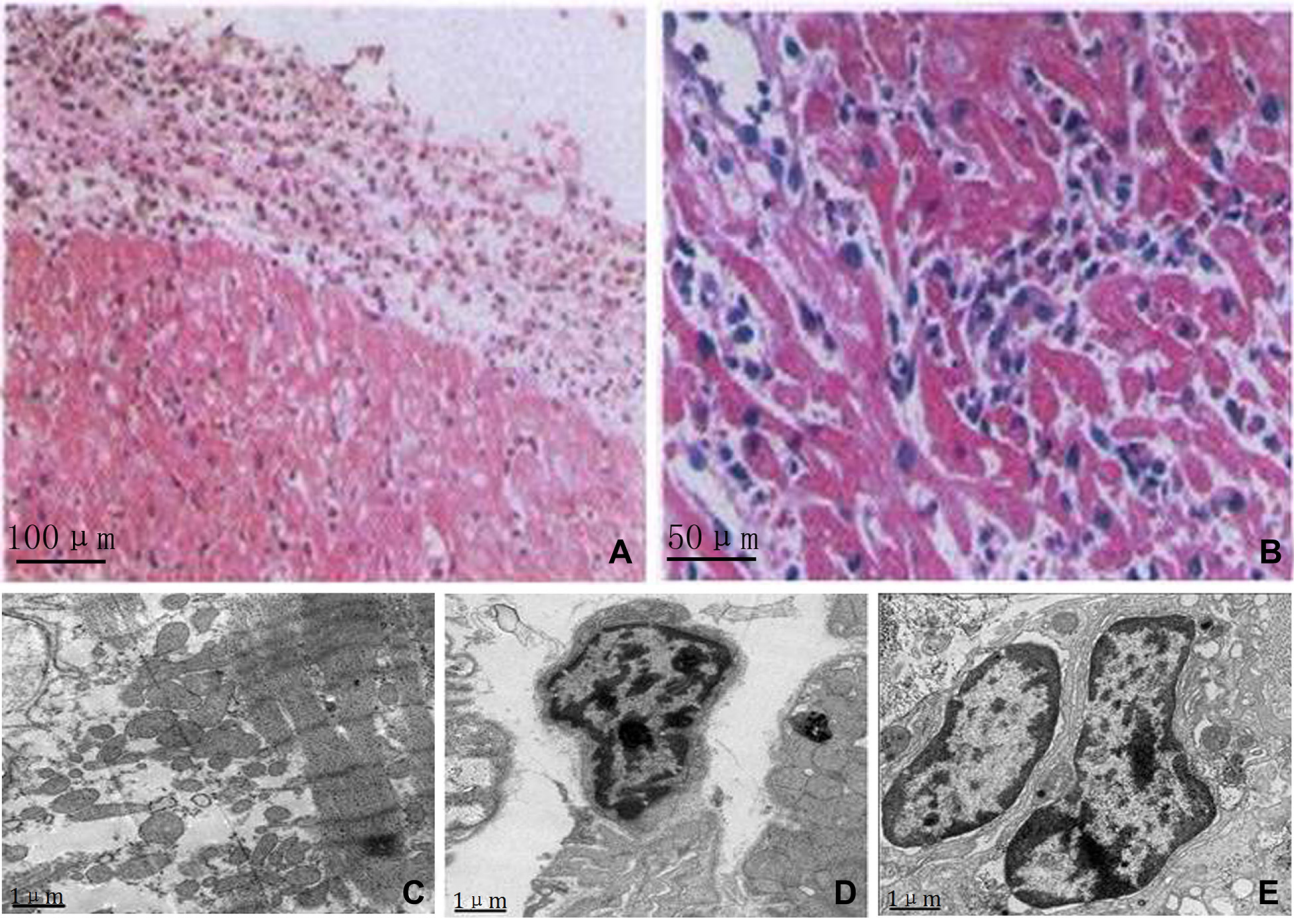
**Endomyocardial biopsy and cardiac electrogram.** Endomyocardial biopsy **(A and B)** showed there was obvious infiltration of EC and focal necrosis. Electron microscopy **(C, D and E)** revealed extensive myocardial interstitial infiltration by EC accompanied by focal myocardial fiber cracking and disintegration Internal scales: **A**: 100 μm; **B**: 50 μm; **C**, **D** and **E**: 1 μm.

Based on these findings, with particular reference to the significant increase in the peripheral eosinophil count in the present case, a diagnosis of systemic disease resulting in focal eosinophilic infiltration and severe myocardial damage was initially postulated. To confirm the final diagnosis, the patient underwent an EMB, which showed obvious diffuse EC infiltration without focal necrosis (Figure 5). Electron microscopy exhibited extensive myocardial interstitial infiltration by EC accompanied by focal myocardial fiber cracking and disintegration (Figure 5).

At 11:00 pm on the March 15, 2012, the patient presented with chest tightness, fatigue, irritability, fever and chills, with a body temperature of 39°C. Subsequently, his blood pressure declined, followed by respiratory and cardiac arrest. After immediate chest compression, defibrillation and tracheal intubation, the patient’s circulation was recovered successfully.

On March16, 2012, methylprednisolone pulse therapy (80 mg) was administered and the patient’s EC levels returned to normal on the next day but had risen again to 2.0 × 10^9^/L on the third day. On March 17, 2012, the patient was extubated and conscious and his cardiac function markedly improved. The final diagnosis of eosinophilic myocarditis was confirmed and steroid treatment was started with methylprednisolone pulse therapy (500 mg) daily for the first three days and a maintenance dose of 60 mg for the next seven days. The patient continued to receive a dose of 1 mg/kg/d, which was gradually reduced to 5 mg every two weeks in the long-term follow-up visits. Metoprolol sustained-release tablets (47.5 mg/d), perindopril tablets (2 mg/d) and rimetazidine dihydrochloride tablets (20 mg/q8h) were also administered. On April 25, 2012, the patient was healthy enough to be discharged from the hospital. The follow-up data of the patient showed normalized EC, return of PLT to standard levels and complete recovery of cardiac function.

## Discussion

EM is a rare form of myocarditis characterized pathologically by diffuse focal myocardial inflammation with eosinophilic infiltration, often in association with peripheral blood eosinophilia [4]. In this case, we suspected focal eosinophilic infiltration following CMR imaging and confirmed them by endomyocardial biopsy. We used high-dose corticosteroids to reverse the cardiac injury and to improve the clinical outcome .In the current case presentation, the laboratory features of focal eosinophilic infiltration were observed in both CMR imaging and endomyocardial biopsy. This evidence of marked cardiac damage led to a rapid diagnosis of eosinophilic myocarditis, which was critical for timely and appropriate steroid therapy. This treatment strategy was based on previously documented cases from abroad, which reported that the use of steroids is predictably effective in the early stages to reverse cardiac injury and to improve the prognosis of patients with eosinophilic myocarditis.

Different etiologies for eosinophilic myocarditis have been described including hypereosinophilic syndrome, Loeffler endocarditis and tropical endomyocardial fibrosis. Myocarditis due to hypersensitivity to medication and numerous drugs have been implicated as the most common cause [5]. Eosinophilia is generally secondary to the effects of an array of pathogens, and the usage of tuberculosis drugs such as isoniazid, rifampicin and pyrazinamide have been implicated in previous case reports of EM.

At the cellular level, the mechanism of EM involves activation-stimulated eosinophilic degranulation of a number of compounds, especially eosinophilic cationic proteins that mediate myocardial tissue damage, eventually leading to eosinophilic myocarditis.

According to previous reports [6-8], Cardiac involvement occurs in 54–82% of cases, and, the prognosis is determined by the extent of the endocardial fibrosis and related complications. The five year mortality of EM is 30%.

The proposed mechanism of EM in association with abnormally high EC counts is characterized predominantly by diffuse or focal eosinophilic infiltration of the myocardium. This infiltration plays a vital role in the pathogenesis of eosinophilic myocarditis via release of eosinophilic granule proteins such as ECP and major basic protein (MBP), which causes dysfunction of myocyte mitochondria leading to myocardial lesions [9], as well as endocardial necrosis [10].

Myocarditis presents in many different ways, ranging from mild symptoms of chest pain and palpitations associated with transient ECG changes, to life-threatening cardiogenic shock and ventricular arrhythmia [2]. As an acute inflammatory response with abundant eosinophils, myocytolysis may develop, as well as endomyocardial thrombi, necrotizing vasculitis, pericarditis, pericardial fibrosis and endomyocardial fibrosis [11]. Studies [11,12] have also indicated that cardiac failure, arterial embolism, endomyocardial thickening and left ventricular lateral thrombosis are the most common presentations of eosinophilic myocarditis with cardiac involvement. Cardiac involvement evolves in three stages [13], the first stage being the acute necrotic stage. At this stage, during which most patients are asymptomatic, myocarditis is characterized by cellular infiltration and inflammation as well myocardial necrosis and, in some cases, eosinophilic granulomas [14]. The second or thrombotic stage, also known as the mural thrombus stage, is characterized by the formation of intracardiac mural thrombi, resulting in down-stream thromboembolism. The third and of fibrotic stage is characterized by fibrosis of the thrombus-damaged myocardium. This is the final pathological stage of the disease, which eventually leads to restrictive cardiomyopathy as well as atrioventricular valve dysfunction [15].

Accurate assessment of eosinophilic myocarditis is limited by difficulties in establishing the diagnosis, which is made on the basis of medical history, clinical presentation, and laboratory findings. In some cases, it is difficult to differentiate myocarditis from acute myocardial infarction due to similar presentations and lack of specific evidence; thus, the diagnosis process can impede the timely treatment of eosinophilic myocarditis in the early stages [16]. This is critical for the prevention of the rapid clinical progression and high mortality associated with eosinophilic myocarditis. However, current techniques provide extremely limited ability to establish a definitive diagnosis, as well as to monitor the course of the disease.

The marked elevation of eosinophil counts, although not specific, supports the diagnosis of eosinophilic myocarditis, and some studies [10,17] have indicated that the number of degranulated eosinophils and serum ECP levels can be valuable and objective parameters for monitoring disease activity, particularly after treatment. In addition, coronary angiograms can be used to exhibit thrombus formation and subsequent blockage of the coronary vessels [18].

CMR imaging is reported by Debl et al. [19] as the only noninvasive method that can be used prior to EMB to visualize the extent of endomyocardial involvement in the early diagnosis of eosinophilic myocarditis as well as in the assessment during treatment. Mavrogeni et al. [20] also reported CMR, as an emerging role to evaluate the Kawasaki disease(KD) which is a specific type of systemic vasculitis, can image myocardial inflammation, myocardial perfusion, ventricular function and fibrosis, offering crucial detailed clinical information for diagnosis of EM.

Nuclear imaging, in which endomyocardial fibrosis, edema, and pericardial effusion toward the cardiac apex are frequent findings, is not routinely recommended for the diagnosis of myocarditis, due to its limited availability and risk of radiation exposure [2].

The concept that EMB is the gold standard for a definitive diagnosis of myocarditis is strongly endorsed despite its limited sensitivity and specificity [21,22]. EMB findings are characterized by diffuse myocardial necrosis associated with extensive eosinophilic infiltration of the myocardial interstitium, focal myocyte dissolution, perivascular infiltration and myocardial interstitial fibrosis [23].

The therapy of choice after a diagnosis EM consists of standard heart failure medication and early treatment with high doses of cortisone [24]. The early initiation of steroid therapy can achieve substantial improvements in clinical outcomes, prognosis and long-term survival [25]. In three cases presented by Wong et al. [26], all the patients demonstrated complete recovery and normalization of cardiac contractility after treatment with high-dose oral steroids with gradual tapering. Left ventricular assist device (LVAD) support [27] is a useful option for refractory conditions in cases of heart failure or arrhythmia. This technology has been successfully used to bridge patients with acute myocarditis to recovery as well as in the transition of patients awaiting heart transplantation.

## Conclusion

In summary, myocarditis presents in a variety of ways and EM should be considered in cases with eosinophil elevation. In this case, the results of relevant laboratory analyses and several clinical features correlating with the use of antituberculosis drugs led to the suspicion of EM. This diagnosis was supported by EMB and CMR imaging results. Swift diagnosis with rapid initiation of steroid treatment achieved good clinical effect and complete resolution of EM as well as marked improvement in the patient’s prognosis.

## Consent

The authors declare that they have followed the protocols of the Ethics committee of the First Affiliated Hospital of Wenzhou Medical University (China) in the publication of patient data and that the patient included in the study received sufficient information and gave his written informed consent to participate in the study.
